# The mode of action of the Protein tyrosine phosphatase 1B inhibitor Ertiprotafib

**DOI:** 10.1371/journal.pone.0240044

**Published:** 2020-10-02

**Authors:** Ganesan Senthil Kumar, Rebecca Page, Wolfgang Peti

**Affiliations:** Department of Chemistry and Biochemistry, University of Arizona, Tucson, Arizona, United States of America; George Washington University, UNITED STATES

## Abstract

Protein tyrosine phosphatase 1B (PTP1B) is a validated therapeutic target for the treatment of diabetes and obesity. Ertiprotafib is a PTP1B inhibitor that reached the clinical trial stage for the treatment of diabetes. Interestingly, Ertiprotafib reduces the melting temperature of PTP1B in differential scanning fluorimetry (DSF) assays, different from most drugs that increase the stability of their target upon binding. No molecular data on how Ertiprotafib functions has been published. Thus, to gain molecular insights into the mode of action of Ertiprotafib, we used biomolecular NMR spectroscopy to characterize the molecular details of the PTP1B:Ertiprotafib interaction. Our results show that Ertiprotafib induces aggregation of PTP1B in a concentration dependent manner. This shows that the insufficient clinical efficacy and adverse effects caused by Ertiprotafib is due to its tendency to cause aggregation of PTP1B.

## Introduction

Protein tyrosine phosphorylation is a key post-translational modification that plays essential roles in cell growth, cell differentiation, cell-cycle regulation and the immune response [1, 2]. The level of protein tyrosine phosphorylation is maintained by the concerted action of protein tyrosine kinases (PTKs; ~90 members) and protein tyrosine phosphatases (PTPs; ~107 members). Aberrant protein tyrosine phosphorylation due to imbalances in the activities of PTKs and PTPs has been implicated in numerous human diseases including cancer and diabetes [3, 4]. Hence, targeting the activities of PTKs and PTPs have emerged as a promising avenue for the development of drugs to cure cancer and diabetes [5–9].

Protein tyrosine phosphatase 1B, the founding member of the PTP superfamily, is critical for the regulation of insulin signaling as it dephosphorylates both the insulin receptor (IR) and the insulin receptor substrate (IRS) [10–12]. Hence, PTP1B is an exceptionally well-studied target for the treatment of diabetes and obesity [13]. We and others have recently shown that PTP1B is a highly regulated enzyme that can be inhibited by either active site or allosteric inhibitors [14, 15]. While its core catalytic functions are driven by rigid conformational changes, allostery in PTP1B is controlled by conformational and dynamic changes, especially in a PTP1B specific secondary structure element, helix α7 [16].

A large number of PTP1B inhibitors have been developed over last two decades [17–19]. Most of these inhibitors target the catalytic site and are mimics of the phosphotyrosine (pTYR) moiety. However, the catalytic site of PTP1B is both conserved and charged. Thus, PTP1B active site inhibitors are often not selective nor are they cell permeable. More recently, a number of allosteric inhibitors have been developed that target an allosteric binding pocket that lies at the intersection PTP1B helices α3, α6 and α7 [15, 20]. Thus far, only two small-molecule PTP1B inhibitors, namely Ertiprotafib [21] and Trodusquemine (also known as MSI-1436) [22] have successfully reached clinical trials. We and others have recently shown that Trodusquemine inhibits PTP1B activity in a unique fashion, namely by binding to the intrinsically disordered C-terminus of PTP1B, demonstrating it functions in an allosteric manner [15].

**Fig 1:**
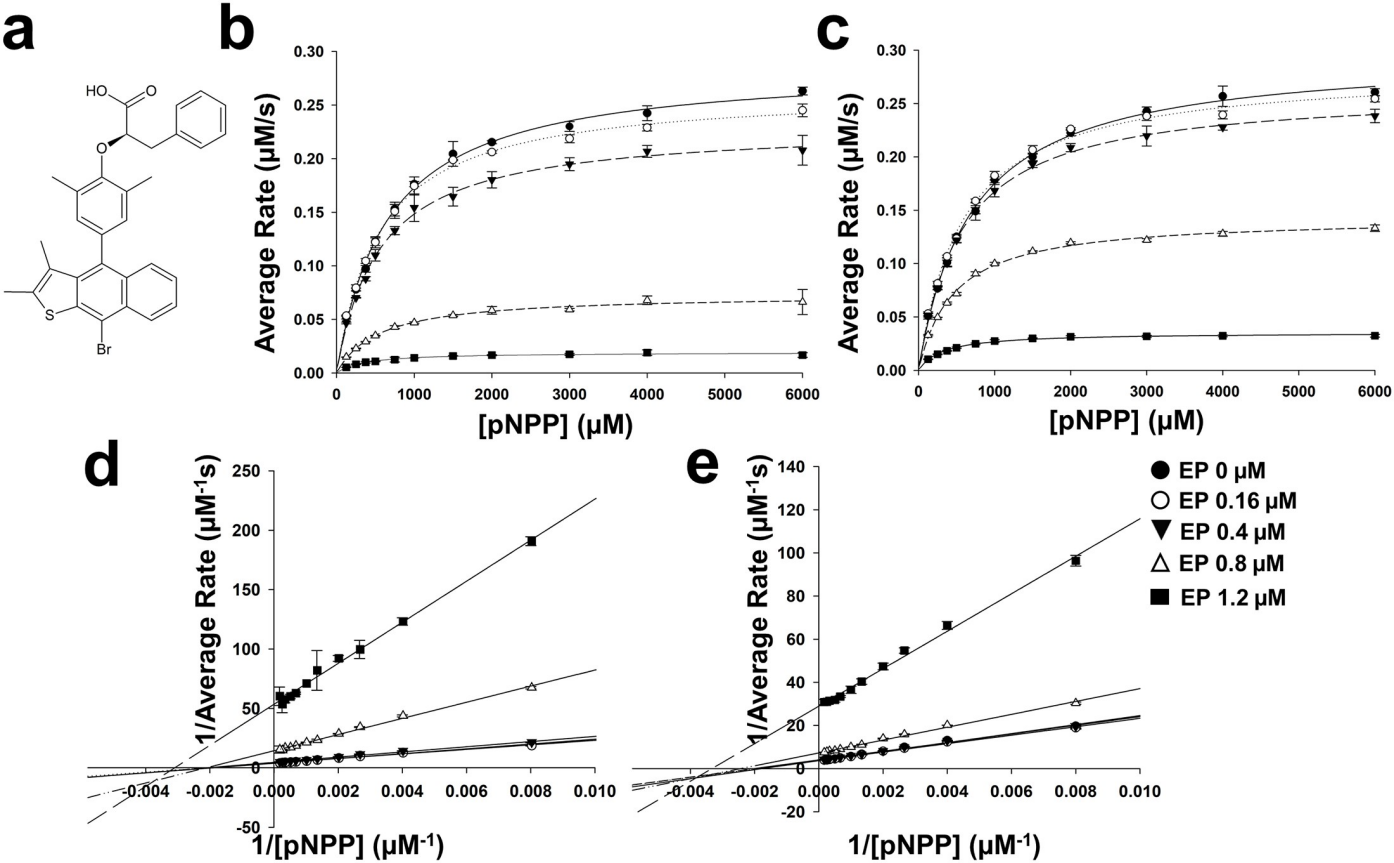
Ertiprotafib is a non-competitive inhibitor of PTP1B. (a) Chemical structure of Ertiprotafib. Michaelis-Menten kinetics of (b) PTP1B^1-301^ and (c) PTP1B^1-393^ as a function of Ertiprotafib concentration using pNPP as substrate. Note the decrease in activity of PTP1B as a function of Ertiprotafib concentration. Lineweaver–Burk plot of (d) PTP1B^1-301^and (e) PTP1B^1-393^ showing the mode of Ertiprotafib inhibition.

Ertiprotafib is a monocarboxylic acid pTyr mimetic and thus it was assumed that it binds at the PTP1B active site (Fig 1a). Interestingly, Ertiprotafib inhibits both PTP1B and the dual peroxisome proliferator-activated receptor (PPAR) alpha and gamma agonist with hypoglycemic and anti-lipidemic activity [23]. Ertiprotafib was in phase II clinical studies for the treatment of type 2 diabetes in both the USA and Europe, but was discontinued due to unsatisfactory phase II clinical efficacy and dose-limiting adverse effects in some patients [21]. Although Ertiprotafib is no longer under investigation, it might provide a platform for the development of other PTP1B inhibitors based on its affinity, acceptable selectivity and oral availability. Strikingly, in differential scanning fluorimetry (DSF) assays, which are commonly used as primary drug screen, Ertiprotafib lowered the melting temperature of PTP1B [24, 25]. This is different to most drugs that bind tightly to their targets, resulting in an increase in their melting temperatures. This suggests Ertiportafib may have an atypical mode-of-action for PTP1B inhibition. We set out to determine the molecular basis for the Ertiprotafib interaction with PTP1B in order to determine if further development of this class of inhibitor is warranted.

Here we use solution state bimolecular NMR spectroscopy to determine the mode of binding to and inhibition of PTP1B by Ertiprotafib. Unexpectedly, our data show that Ertiprotafib binds non-specifically to the catalytic domain of PTP1B, ultimately leading to PTP1B aggregation. Thus, Ertiprotafib inhibits the activity of PTP1B by inducing aggregation and not by either directly blocking the active site or inhibiting PTP1B allosterically. This mode-of-action also explains the reduction of the PTP1B melting temperature and highlights that destabilizers identified in DSF screens should be tested for their molecular modes-of-action prior to extensive efforts for compound optimization.

## Materials and methods

### Protein production

DNA coding the human PTP1B catalytic domain (residues 1–301; PTP1B^1-301^) and PTP1B catalytic domain with the C-terminus disordered tail (residues 1–393; PTP1B^1-393^) were sub-cloned into RP1B as described [15, 26]. For protein expression, plasmid DNAs were transformed into *E*. *coli* BL21 (DE3) RIL cells (Agilent). Cells were grown in Luria Broth in the presence of selective antibiotics at 37°C to an OD_600_ of ~0.8–1.0 and expression was induced with the addition of 1 mM isopropyl-β-D-1-thiogalactopyranoside (IPTG, GoldBIO). Cells were grown for additional 18–20 hours at 18°C and harvested by centrifugation at 6000 *x*g. Cell pellets were stored at -80°C until purification. For NMR measurements, expression of uniformly [^2^H,^15^N] labeled PTP1B was achieved by growing cells in D_2_O based M9 minimal media containing 1g/L ^15^NH_4_Cl (CIL) as the sole nitrogen source. Multiple rounds (0%, 30%, 50%, 70% and 100%) of D_2_O adaptation were necessary for high-yield expression [27].

Cell pellets were resuspended in Lysis Buffer (50 mM Tris pH 8.0, 500 mM NaCl, 5 mM imidazole, 0.1% Triton X-100) containing EDTA-free protease inhibitor cocktail tablets (Roche) and lysed using a high-pressure homogenization (Avestin). The bacterial lysate was clarified by centrifugation at 45,000 *x*g and filtered through a 0.22 μm filter before loading onto a His-trap HP column (GE Healthcare). Bound proteins were washed with Buffer A (50 mM Tris pH 8.0, 500 mM NaCl, 5 mM imidazole) and eluted with increasing amounts of Buffer B (50 mM Tris pH 8.0, 500 mM NaCl, 500 mM imidazole) using a 5–500 mM imidazole gradient. Peak fractions were pooled and dialyzed overnight at 4°C in Dialysis Buffer (50 mM Tris pH 8.0, 500 mM NaCl) with 5:1 volume ratio of TEV protease overnight. The next day, a subtraction His_6_-tag purification was performed to remove the TEV protease and the cleaved His_6_-tag. Cleaved PTP1B was concentrated and further purified using Size Exclusion Chromatography (SEC, Superdex 75 26/60 [GE Healthcare]) in Assay/DLS/NMR Buffer (10 mM HEPES pH 7.4, 150 mM NaCl, 0.5 mM TCEP). Purified PTP1B was either used immediately or flash frozen in liquid nitrogen for storage. Typical PTP1B protein yields were ~ 45 mg/L in Luria Broth and ~35 mg/L in ^2^H/^15^N M9 medium.

### NMR spectroscopy

All NMR experiments were acquired at 298 K on Bruker Avance NEO 800 MHz ^1^H Larmor frequency NMR spectrometer equipped with a TCI-active HCN-active z-gradient cryoprobe. The interaction between PTP1B^1-301^ and PTP1B^1-393^ with Ertiprotafib was studied by direct comparison of 2D [^1^H,^15^N] TROSY spectra of free and Ertiprotafib bound [^2^H,^15^N]-labeled PTP1B^1-301/1-393^. Ertiprotafib was titrated into [^2^H,^15^N]-labeled PTP1B^1-301/1-393^ at increasing molar ratios (one, two, five, eight, ten and fifteen) to monitor the chemical shift perturbation and intensity changes as a function of Ertiprotafib concentration. Ertiprotafib was solubilized in DMSO-d_6_ (no intensity changes were observed in a 2D [^1^H,^15^N] TROSY spectrum of PTP1B when adding DMSO alone). The final concentration of PTP1B was 0.15 mM in 10 mM HEPES pH 7.4, 150 mM NaCl, 0.5 mM TCEP, and 90% H_2_O/10% D_2_O. The spectra were processed using Topspin 4.0.3 (Bruker, Billerica, MA) and analyzed using NMRFAM-Sparky [28]. Backbone amide chemical shift deviations were calculated using the formula: Δδ_av_ = sqrt(0.5((δ_HN, bound_-δ_HN,free_)^2^ + 0.04 (δ_N,bound_-δ_N,free_)^2^)). The sequence specific backbone assignments of PTP1B^1-301^ and PTP1B^1-393^ were reported earlier [15].

### DLS measurements

Dynamic light scattering (DLS) data for PTP1B^1-301^ and PTP1B^1-393^ with increasing molar ratios of Ertiprotafib (0, 2, 5, 10 and 15) were acquired using a Wyatt DynaPro Nanostar. DLS size distribution histograms were calculated with DYNAMICS 6.12 (Wyatt Technology Corporation). All of the proteins were measured in triplicate at a concentration of 1 mg/ml in 10 mM HEPES pH 7.4, 150 mM NaCl, 0.5 mM TCEP. Each experiment reflects the average of ten measurements, each of which was acquired for 10 s at 25 °C.

### Protein stability measurements

Melting temperature (T_m_) measurements that report PTP1B stability as a function of increasing concentrations of Ertiprotafib (in 10 mM HEPES pH 7.4, 150 mM NaCl, 0.5 mM TCEP; 1 mg/ml; 3 replicates; increasing molar ratios of Ertiprotafib of 0, 2, 5, 10 and 15) were performed on a Tycho NT.6 (Nanotemper) using standard capillaries (10 μl) using a 30 °C/min ramp (from 35 to 95 °C) and evaluated using the Tycho NT.6 software version 1.1.5.668.

### pNPP activity assay

The activities of PTP1B^1-301^ and PTP1B^1-393^ were measured in assay buffer containing varying concentrations of p-nitrophenyl phosphate (pNPP; 0 to 6 mM final concentrations) with increasing concentrations of Ertiprotafib (0 μM, 0.16 μM, 0.4 μM, 0.8 μM and 1.2 μM). Enzymes were incubated with the substrate at 30 °C for 30 min. The reaction was stopped using 1 M NaOH and the absorbance was measured at 405 nM using a plate reader (BioTek). Ertiprotafib stock solution (25 mM) was prepared in 100% DMSO, resulting in 0.1–0.8% of DMSO in the reactions. Measured absorbance from blanks that contained substrate and inhibitors but no protein was subtracted from all measurements. The rate of dephosphorylation of pNPP was analyzed using the molar extinction coefficient for pNPP of 18000 M^-1^cm^-1^ and an optical length of 0.3cm (96 well plates). K_m_ and V_max_ were determined by fitting to the Michaelis-Menten equation, y = V_max_*x/(K_m_+x); k_cat_ was extracted using y = E_t_*k_cat_. The catalytic efficiency was obtained as k_cat/_K_m_. Sigmaplot 13.0 was used for data analysis. The experiments were carried out in triplicate.

## Results

### Ertiprotafib is a non-competitive inhibitor of PTP1B

Ertiprotafib was initially identified as a potent inhibitor of PTP1B (435 residues; ~50 kDa; the C-terminal ~30 aa form an ER targeting α-helix). To test the activity Ertiprotafib against different PTP1B constructs, we performed activity assays using p-nitrophenyl phosphate (pNPP) as the PTP1B substrate. Specifically, we used two constructs of PTP1B that we used previously to establish the mode of action of Trodusquemine/MSI-1436 [15]: (1) the catalytic domain of PTP1B, PTP1B^1-301^ (~36 kDa; aa 1–301) and (2) PTP1B^1-393^ (~45 kDa; aa 1–393), which includes PTP1B^1-301^ and its intrinsically disordered C-terminal tail, PTP1B^302-393^. We recently showed that the C-terminal tail allosterically modulates the activity of PTP1B and is the key binding site of MSI-1436/Trodusquemine [15]. At low Ertiprotafib concentrations (< 0.4 μM), v_max_ is decreased whereas K_M_ remained largely unchanged, showing that Ertiprotafib is a non-competitive inhibitor of PTP1B (Fig 1b–1e, Table 1). However, at higher Ertiprotafib concentrations (>0.4 μM), PTP1B showed reduced activity (>50%) with lower v_max_ and K_M_ values, thereby exhibiting mixed inhibition. This suggests PTP1B inhibition by Ertiprotafib exhibits non-classical enzyme kinetics. Finally, because PTP1B^1-301^ and PTP1B^1-393^ show identical inhibition profiles, the C-terminal IDR tail of PTP1B does not contribute to the mode of action of Ertiprotafib.

**Table 1:** Turnover rates (k_cat_) and catalytic efficiencies (k_cat_/K_m_) of PTP1B in the presence of Ertiprotafib.

	k_cat_(s^-1^)	K_M_	v_max_	fold	k_cat_/K_m_ (x10^3^ M^-1^ s^-1^)	R^2^	N
**PTP1B**^**1-301**^	3.6 ± 0.2	662 ± 24	0.29 ± 0.003	1.0	5.4 ± 0.4	0.993	3
+ 0.16 μM Ertiprotafib	3.3 ± 0.2	556 ± 18	0.26 ± 0.003	0.8	5.9 ± 0.4	0.993	3
+ 0.4 μM Ertiprotafib	3.0 ± 0.2	555 ± 28	0.23 ± 0.003	0.8	5.2 ± 0.4	0.984	3
+ 0.8 μM Ertiprotafib	0.9 ± 0.1	548 ± 28	0.07 ± 0.001	0.8	1.7 ± 0.1	0.958	3
+ 1.2 μM Ertiprotafib	0.2 ± 0.1	355 ± 38	0.02 ± 0.001	0.5	0.7 ± 0.1	0.914	3
**PTP1B**^**1-393**^	3.7 ± 0.2	716 ± 22	0.30 ± 0.003	1.0	5.2 ± 0.3	0.995	3
+ 0.16 μM Ertiprotafib	3.5 ± 0.2	588 ± 19	0.28 ± 0.003	0.8	6.0 ± 0.4	0.994	3
+ 0.4 μM Ertiprotafib	3.3 ± 0.2	568 ± 17	0.26 ± 0.002	0.8	5.8 ± 0.4	0.995	3
+ 0.8 μM Ertiprotafib	1.8 ± 0.1	460 ± 12	0.14 ± 0.001	0.6	3.9 ± 0.2	0.995	3
+ 1.2 μM Ertiprotafib	0.4 ± 0.1	320 ± 14	0.04 ± 0.001	0.5	1.4 ± 0.1	0.984	3

### Molecular basis of PTP1B:Ertiprotafib interaction

**Fig 2:**
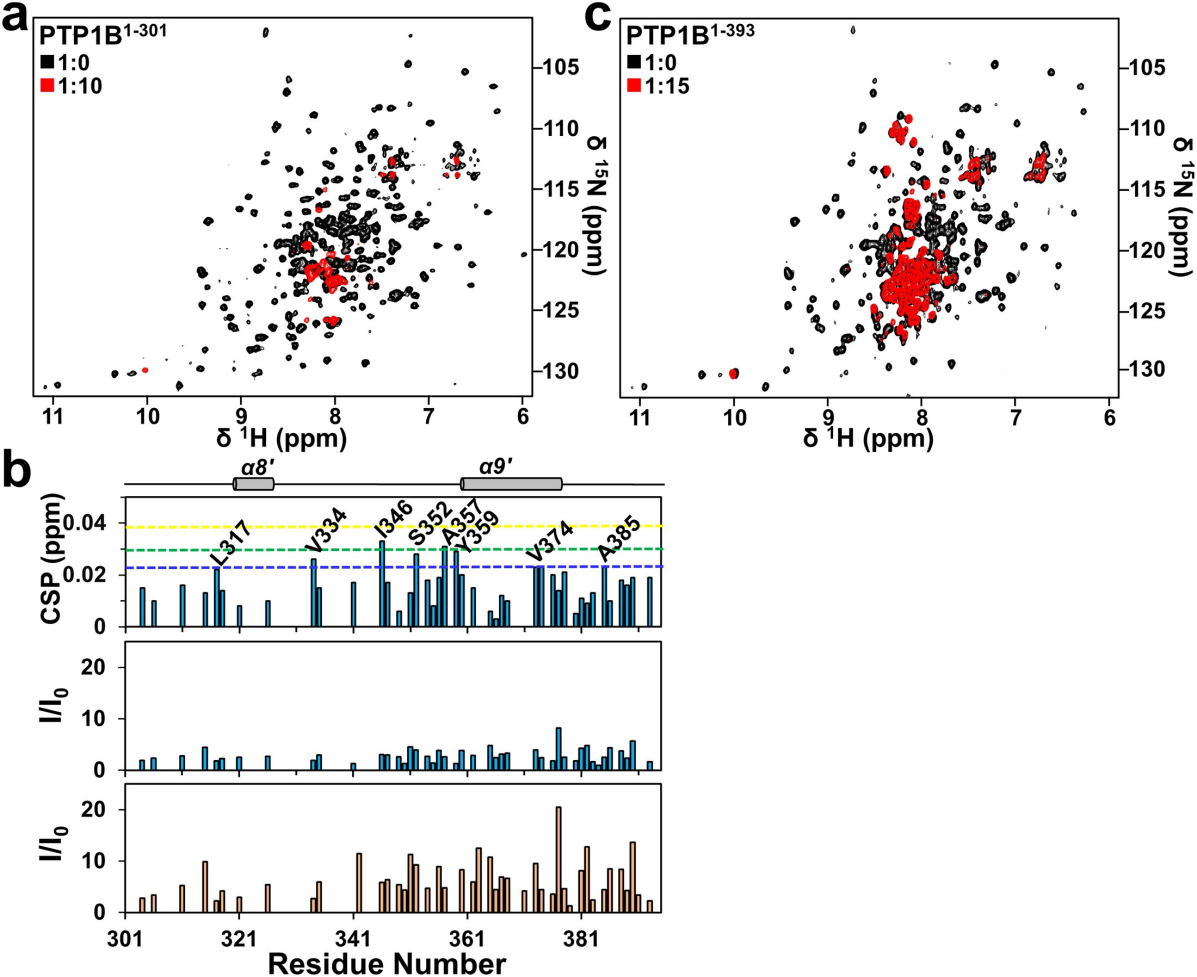
Ertiprotafib binds PTP1B non-specifically and induces its aggregation. Overlay of the 2D [^1^H,^15^N]-TROSY spectra of (a) PTP1B^1-301^ and (c) PTP1B^1-393^ in the presence of ten and fifteen, respectively, molar equivalents of Ertiprotafib showing the near-complete disappearance of the peaks from the structured regions. (b) Chemical shift (*top panel*) and intensity (*middle panel*, colored in blue) changes of Ertiprotafib binding (at a molar ratio of 10) to PTP1B residues 301–393, illustrating the non-specific interaction of C-terminal disordered region much like another allosteric inhibitor, MSI-1436. Colored lines indicate one (blue), two (green) and three (yellow) s.d. from the mean CSPs. Note the large increase in intensities of the peaks of the C-terminal disordered regions at higher molar ratios of Ertiprotafib (15 molar excess, *bottom panel*, colored in orange).

Our inhibition data is inconsistent with previously published data that suggests Ertiprotafib is an active site inhibitor (IC_50_ >20 μM) of PTP1B [23]. The main motivation for this mode of action was computational docking of Ertiprotafib to a 3D structure of the catalytic domain of PTP1B [29]. Thus, we used biomolecular NMR spectroscopy to experimentally determine the molecular mode of the Ertiprotafib:PTP1B interaction. Unexpectedly, a direct comparison of the 2D [^1^H,^15^N] TROSY spectrum of free and Ertiprotafib-bound PTP1B^1-301^ revealed only very small non-statistically meaningful chemical shift perturbations (CSPs; direct indicator of changes in chemical environment upon binding; Δδ_av_ = 0.01 ± 0.01 ppm; S1a and S1b Fig; ***top panel***). This was surprising as other PTP1B inhibitors, such as TCS-401 or CPT157633, show large, statistically significant CSPs in residues that define the PTP1B active site [14, 16]. Furthermore, these minor CSPs did not change in magnitude with increasing concentrations of Ertiprotafib, as would be expected for a specific interaction. Finally, multiple PTP1B peaks in the 2D [^1^H,^15^N] TROSY spectrum showed line broadening upon addition of Ertiprotafib and this behavior became more and more apparent at higher PTP1B:Ertiprotafib molar ratios. In particular, at high ratios (1:10), nearly all the peaks of PTP1B^1-301^ disappeared (Fig 2a). The simplest interpretation of this data is that PTP1B^1-301^ aggregates upon the addition of increasing amounts of Ertiprotafib.

### The PTP1B C-terminal tail does not contribute to the action of Ertiprotafib

We repeated these NMR experiments using PTP1B^1-393^. As for PTP1B^1-301^ only minor CSPs were detected (S1b Fig**, *bottom panel*** and S1c Fig). Further, a small number of residues in the IDR region showed CSPs including L317, V334, I346, S352, A357, Y359, V374 and A385 (Fig 2b, ***top panel***). As these are mostly hydrophobic residues, we speculated that these might be very weak non-specific interactions. Consistent with the data for PTP1B^1-301^, at high Ertiprotafib ratios (1:15), all peaks belonging to PTP1B^1-301^ residues disappeared (Fig 2c). Residues of the PTP1B IDR C-terminus (residues 302–393) showed increased intensity upon binding to Ertiprotafib (Fig 2b; ***middle and bottom panel***). This increase in intensity for residues in the IDR C-terminal tail shows that this disordered region is highly dynamic and behaves as an independent moiety from the PTP1B catalytic domain, resulting in faster tumbling and higher intensity peaks than those of the PTP1B catalytic domain. Together, these data show that Ertiprotafib induces aggregation of PTP1B^1-301^ independent of the IDR C-terminus and, further, that the nonspecific interaction of Ertiprotafib with the C-terminal residues is not important for Ertiprotafib function. This contrasts with our study of PTP1B with Trodusquemine, where we did not see aggregation when we performed similar experiments [15].

### Ertiprotafib induced aggregation of the PTP1B catalytic domain

**Fig 3:**
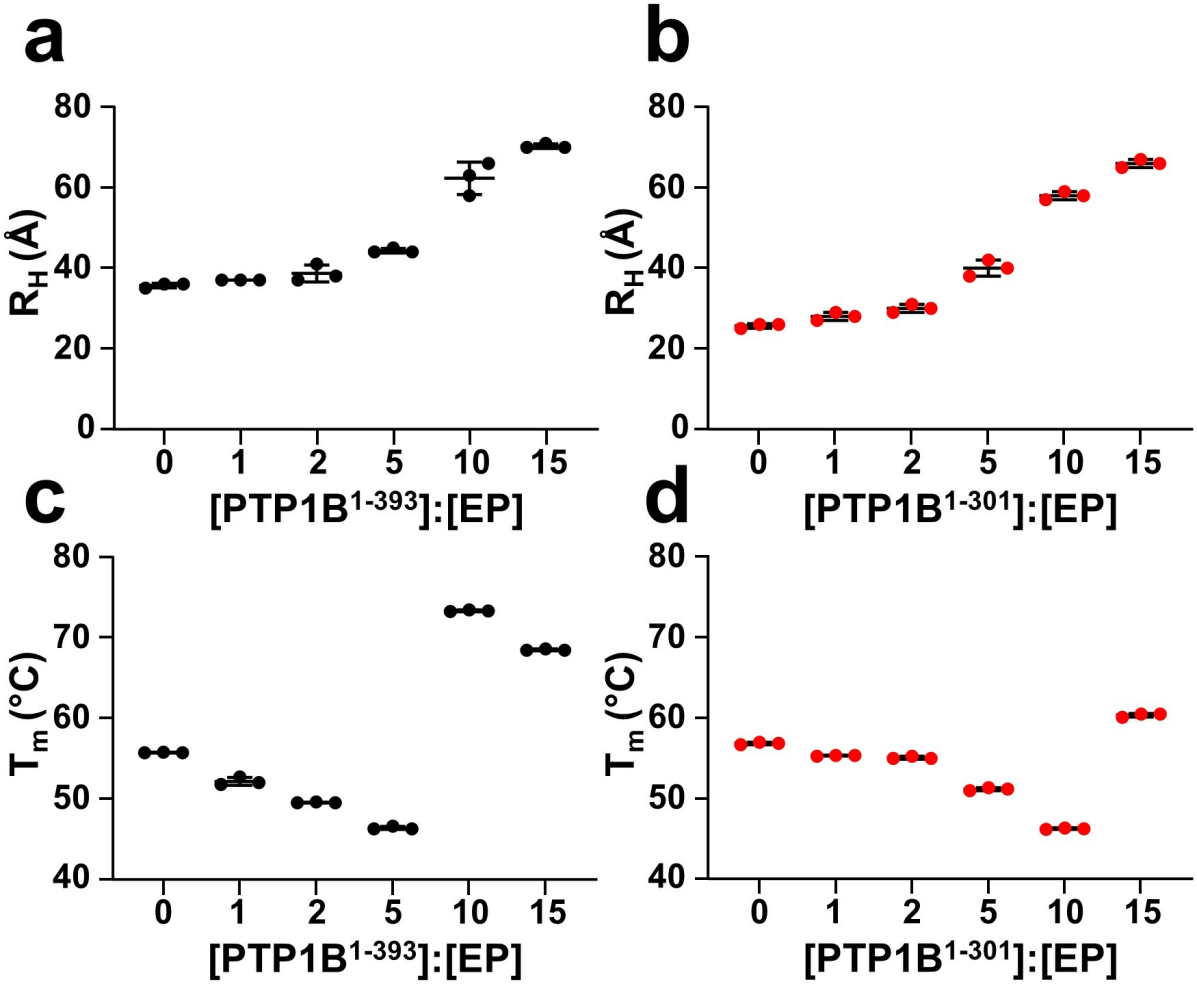
Oligomerization of PTP1B is dependent on Ertiprotafib concentration. Changes in R_H_ of (a) PTP1B^1-393^ (black) and (b) PTP1B^1-301^ (red) as a function of increasing Ertiprotafib concentration as measured by Dynamic light scattering experiments. Note the increase in R_H_ at higher Ertiprotafib concentrations confirms PTP1B aggregation. Melting temperatures (T_m_) of (c) PTP1B^1-393^ (black) and (d) PTP1B^1-301^ (red) as a function of Ertiprotafib concentration.

To confirm that PTP1B^1-301^ and PTP1B^1-393^ aggregate upon Ertiprotafib binding, we used dynamic light scattering (DLS). DLS measurements report the radius of hydration (R_H_) of 25.7 Å ± 0.6 Å and 35.7 Å ± 0.6 Å for PTP1B^1-301^ and PTP1B^1-393^, respectively, consistent with previously reported data [15]. However, PTP1B^1-301^ and PTP1B^1-393^ showed exponential increases in their radii of gyration with increasing concentrations of Ertiprotafib (Fig 3a and 3b). These data not only confirm that PTP1B aggregates in the presence of Ertiprotafib but also that the size of the aggregates increases with increasing concentrations of compound. This behavior was confirmed by measuring the melting temperature (T_m_) of PTP1B in the presence of differing concentrations of Ertiprotafib (Ertiprotafib is known as a ‘destabilizer’ in DSF screens). Different to commonly used DSF measurements, our measurements relied on intrinsic tryptophan fluorescence and not a hydrophobic dye that might lead to aggregation. At low Ertiprotafib concentrations, the T_m_ decreases, similar to reported DSF data. In contrast, at high Ertiprotafib concentrations, the T_m_ increases (Fig 3c and 3d). This is consistent with the fact that Ertiprotafib decreases the stability of PTP1B at lower concentrations but becomes increasingly stable as a result of extensive aggregation.

## Discussion

Ertiprotafib is the first PTP1B inhibitor that made it into clinical trials. In DSF measurements, experiments commonly used in high throughput drug screening assays to identify compounds that bind protein targets, Ertiprotafib was discovered to be a ‘destabilizer’, as it decreased PTP1B thermal stability (T_m_). This is in contrast to most inhibitors/drugs which increase the T_m_ of their protein targets. To define the molecular basis of these observations, we used biomolecular NMR spectroscopy. Our data showed unequivocally that Ertiprotafib induces oligomerization/aggregation of PTP1B. It also showed, mechanistically, how this aggregation is achieved. Namely, Ertiprotafib induces aggregation of the catalytic domain of PTP1B, with the regulatory, intrinsically disordered C-terminal tail playing no significant role in this process. This contrasts with another small molecule inhibitor of PTP1B, MSI-1436, which binds specifically to the disordered C-terminal tail of PTP1B and does not lead to PTP1B aggregation [15]. Thus, of the PTP1B inhibitors molecularly characterized, the aggregation of PTP1B is unique and specific to Ertiprotafib.

During the last two decades, multiple studies have shown that at least a subset of compounds identified primarily via high-throughput screening also ‘inhibit’ by aggregating their target proteins. For example, small molecular inhibitors that target β-lactamase, malarial protease, the insulin receptor and farnesyl transferases, among others, have also been shown to aggregate in solution and it is the aggregation that indirectly results in enzyme inhibition [30, 31]. Our data clearly show that the failure of Ertiprotafib in clinical trials is due to its ability to aggregate PTP1B and hence, its failure to manipulate insulin signaling in a specific, PTP1B-dependent manner. Together, these results suggest that compounds identified to lower the target thermal stability, as observed in the presence of low concentrations of Ertiprotafib, should be tested for their molecular mode of interaction prior to extensive efforts for compound optimization.

## Supporting information

S1 Fig Ertiprotafib induces minor chemical shift changes upon binding to PTP1B. Overlay of the 2D [1H,15N]-TROSY spectra of (a) PTP1B^1-301^ and (c) PTP1B^1-393^ with increasing amounts of Ertiprotafib illustrating the changes in peak intensities. (b) Chemical shift perturbations (CSPs) observed for PTP1B^1-301^ (5 molar excess of Ertiprotafib, top panel) and PTP1B^1-393^ (ten molar excess of Ertiprotafib, bottom panel), respectively.
(DOCX)

## References

[1] Alonso A, Sasin J, Bottini N, Friedberg I, Friedberg I, Osterman A, et al Protein tyrosine phosphatases in the human genome. Cell. 2004;117: 699–711. 10.1016/j.cell.2004.05.018 15186772

[2] Hunter T. The genesis of tyrosine phosphorylation. Cold Spring Harb Perspect Biol. 2014;6: a020644 10.1101/cshperspect.a020644 24789824 PMC3996475

[3] Cohen P. The role of protein phosphorylation in human health and disease. The Sir Hans Krebs Medal Lecture. Eur J Biochem. 2001;268: 5001–5010. 10.1046/j.0014-2956.2001.02473.x 11589691

[4] Hendriks WJAJ, Elson A, Harroch S, Pulido R, Stoker A, den Hertog J. Protein tyrosine phosphatases in health and disease. FEBS J. 2013;280: 708–730. 10.1111/febs.12000 22938156

[5] Roskoski R. A historical overview of protein kinases and their targeted small molecule inhibitors. Pharmacol Res. 2015;100: 1–23. 10.1016/j.phrs.2015.07.010 26207888

[6] Stanford SM, Bottini N. Targeting Tyrosine Phosphatases: Time to End the Stigma. Trends Pharmacol Sci. 2017;38: 524–540. 10.1016/j.tips.2017.03.004 28412041 PMC5494996

[7] Tonks NK. Protein tyrosine phosphatases—from housekeeping enzymes to master regulators of signal transduction. FEBS J. 2013;280: 346–378. 10.1111/febs.12077 23176256 PMC3662559

[8] Krishnan N, Bonham CA, Rus IA, Shrestha OK, Gauss CM, Haque A, et al Harnessing insulin- and leptin-induced oxidation of PTP1B for therapeutic development. Nat Commun. 2018;9: 283 10.1038/s41467-017-02252-2 29348454 PMC5773487

[9] He R-J, Yu Z-H, Zhang R-Y, Zhang Z-Y. Protein tyrosine phosphatases as potential therapeutic targets. Acta Pharmacol Sin. 2014;35: 1227–1246. 10.1038/aps.2014.80 25220640 PMC4186993

[10] Tonks NK, Diltz CD, Fischer EH. Purification of the major protein-tyrosine-phosphatases of human placenta. J Biol Chem. 1988;263: 6722–6730. 2834386

[11] Tonks NK, Diltz CD, Fischer EH. Characterization of the major protein-tyrosine-phosphatases of human placenta. J Biol Chem. 1988;263: 6731–6737. 2834387

[12] Goldstein BJ, Bittner-Kowalczyk A, White MF, Harbeck M. Tyrosine dephosphorylation and deactivation of insulin receptor substrate-1 by protein-tyrosine phosphatase 1B. Possible facilitation by the formation of a ternary complex with the Grb2 adaptor protein. J Biol Chem. 2000;275: 4283–4289. 10.1074/jbc.275.6.4283 10660596

[13] Feldhammer M, Uetani N, Miranda-Saavedra D, Tremblay ML. PTP1B: a simple enzyme for a complex world. Crit Rev Biochem Mol Biol. 2013;48: 430–445. 10.3109/10409238.2013.819830 23879520

[14] Krishnan N, Krishnan K, Connors CR, Choy MS, Page R, Peti W, et al PTP1B inhibition suggests a therapeutic strategy for Rett syndrome. J Clin Invest. 2015;125: 3163–3177. 10.1172/JCI80323 26214522 PMC4563751

[15] Krishnan N, Koveal D, Miller DH, Xue B, Akshinthala SD, Kragelj J, et al Targeting the disordered C terminus of PTP1B with an allosteric inhibitor. Nat Chem Biol. 2014;10: 558–566. 10.1038/nchembio.1528 24845231 PMC4062594

[16] Choy MS, Li Y, Machado LESF, Kunze MBA, Connors CR, Wei X, et al Conformational Rigidity and Protein Dynamics at Distinct Timescales Regulate PTP1B Activity and Allostery. Mol Cell. 2017;65: 644–658.e5. 10.1016/j.molcel.2017.01.014 28212750 PMC5325675

[17] He R, Zeng L-F, He Y, Zhang S, Zhang Z-Y. Small molecule tools for functional interrogation of protein tyrosine phosphatases. FEBS J. 2013;280: 731–750. 10.1111/j.1742-4658.2012.08718.x 22816879 PMC3495087

[18] Combs AP. Recent advances in the discovery of competitive protein tyrosine phosphatase 1B inhibitors for the treatment of diabetes, obesity, and cancer. J Med Chem. 2010;53: 2333–2344. 10.1021/jm901090b 20000419

[19] Thareja S, Aggarwal S, Bhardwaj TR, Kumar M. Protein tyrosine phosphatase 1B inhibitors: a molecular level legitimate approach for the management of diabetes mellitus. Med Res Rev. 2012;32: 459–517. 10.1002/med.20219 20814956

[20] Wiesmann C, Barr KJ, Kung J, Zhu J, Erlanson DA, Shen W, et al Allosteric inhibition of protein tyrosine phosphatase 1B. Nat Struct Mol Biol. 2004;11: 730–737. 10.1038/nsmb803 15258570

[21] Zhang Z-Y, Lee S-Y. PTP1B inhibitors as potential therapeutics in the treatment of type 2 diabetes and obesity. Expert Opin Investig Drugs. 2003;12: 223–233. 10.1517/13543784.12.2.223 12556216

[22] Lantz KA, Hart SGE, Planey SL, Roitman MF, Ruiz-White IA, Wolfe HR, et al Inhibition of PTP1B by trodusquemine (MSI-1436) causes fat-specific weight loss in diet-induced obese mice. Obesity (Silver Spring). 2010;18: 1516–1523. 10.1038/oby.2009.444 20075852

[23] Erbe DV, Wang S, Zhang Y-L, Harding K, Kung L, Tam M, et al Ertiprotafib improves glycemic control and lowers lipids via multiple mechanisms. Mol Pharmacol. 2005;67: 69–77. 10.1124/mol.104.005553 15475571

[24] Matulis D, Kranz JK, Salemme FR, Todd MJ. Thermodynamic stability of carbonic anhydrase: measurements of binding affinity and stoichiometry using ThermoFluor. Biochemistry. 2005;44: 5258–5266. 10.1021/bi048135v 15794662

[25] Weber PC, Salemme FR. Applications of calorimetric methods to drug discovery and the study of protein interactions. Curr Opin Struct Biol. 2003;13: 115–121. 10.1016/s0959-440x(03)00003-4 12581668

[26] Peti W, Page R. Strategies to maximize heterologous protein expression in Escherichia coli with minimal cost. Protein Expression and Purification. 2007;51: 1–10. 10.1016/j.pep.2006.06.024 16904906

[27] Peti W, Page R. NMR Spectroscopy to Study MAP Kinase Binding to MAP Kinase Phosphatases In: Pulido R, editor. Protein Tyrosine Phosphatases: Methods and Protocols. New York, NY: Springer New York; 2016 pp. 181–196. 10.1007/978-1-4939-3746-2_11 27514807

[28] Lee W, Tonelli M, Markley JL. NMRFAM-SPARKY: enhanced software for biomolecular NMR spectroscopy. Bioinformatics. 2015;31: 1325–1327. 10.1093/bioinformatics/btu830 25505092 PMC4393527

[29] Liu J-Z, Zhang S-E, Nie F, Yang Y, Tang Y-B, Yin W, et al Discovery of novel PTP1B inhibitors via pharmacophore-oriented scaffold hopping from Ertiprotafib. Bioorg Med Chem Lett. 2013;23: 6217–6222. 10.1016/j.bmcl.2013.10.002 24148325

[30] McGovern SL, Helfand BT, Feng B, Shoichet BK. A Specific Mechanism of Nonspecific Inhibition. J Med Chem. 2003;46: 4265–4272. 10.1021/jm030266r 13678405

[31] McGovern SL, Caselli E, Grigorieff N, Shoichet BK. A common mechanism underlying promiscuous inhibitors from virtual and high-throughput screening. J Med Chem. 2002;45: 1712–1722. 10.1021/jm010533y 11931626

