# Supplementary material for: The mode of action of the Protein tyrosine phosphatase 1B inhibitor Ertiprotafib
Source: PLoS One. 2020 Oct 2;15(10):e0240044. doi: 10.1371/journal.pone.0240044 (PMC7531832; doi:10.1371/journal.pone.0240044)
Supplement: S1 Fig — Overlay of the 2D [1H,15N]-TROSY spectra of (a) PTP1B1-301 and (c) PTP1B1-393 with increasing amounts of Ertiprotafib illustrating the changes in peak intensities. (b) Chemical shift perturbations (CSPs) observed for PTP1B1-301 (5 molar excess of Ertiprotafib, top panel) and PTP1B1-393 (ten molar excess of Ertiprotafib, bottom panel), respectively. (DOCX) [file pone.0240044.s001.docx]

**The mode of action of the Protein tyrosine phosphatase 1B inhibitor Ertiprotafib**

Ganesan Senthil Kumar^1^, Rebecca Page^1^ and Wolfgang Peti^1,*^


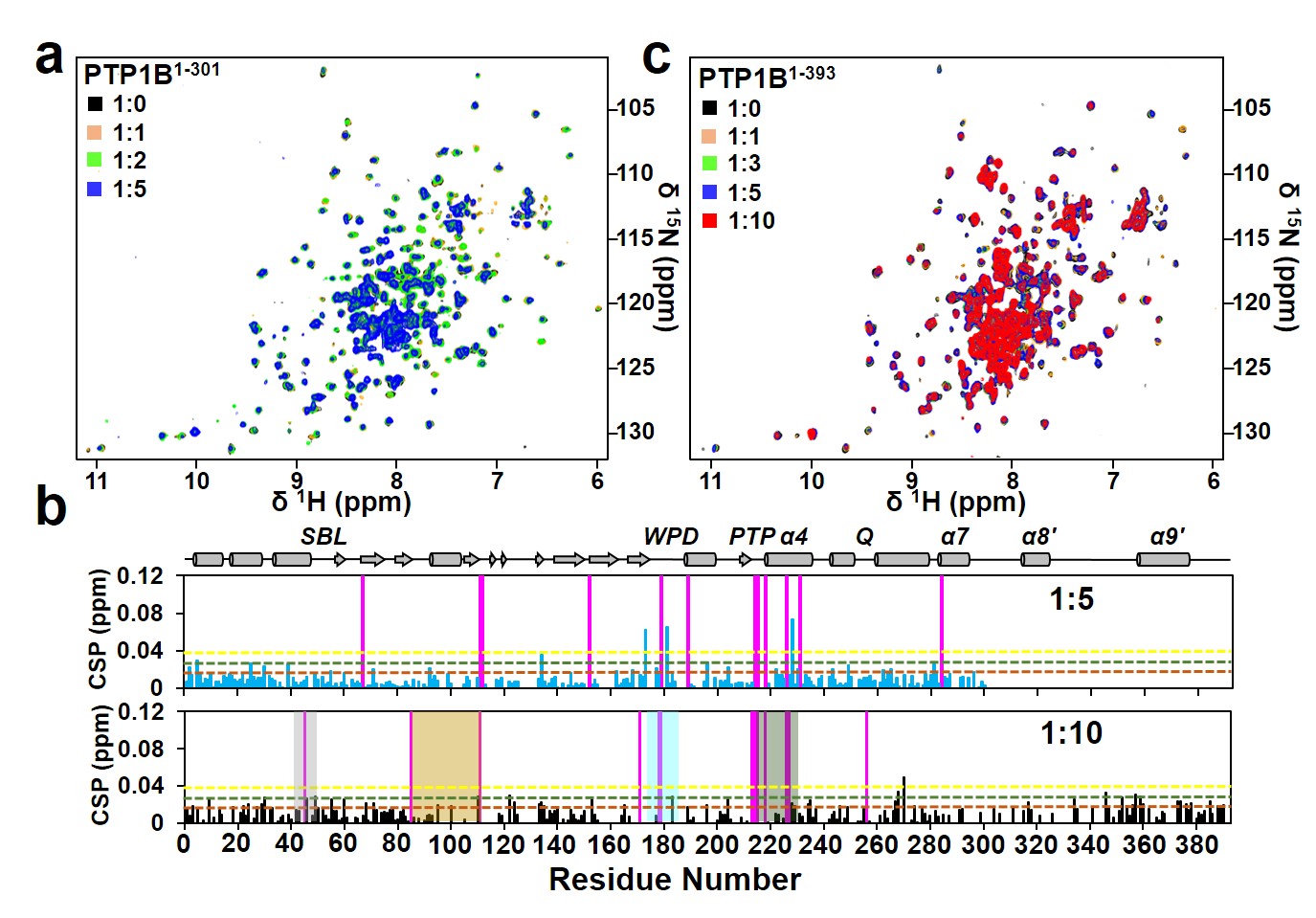


**Supplementary Figure 1.** *Ertiprotafib induces minor chemical shift changes upon binding to PTP1B.* Overlay of the 2D [^1^H,^15^N]-TROSY spectra of (a) PTP1B^1-301^ and (c) PTP1B^1-393^ with increasing amounts of ertiprotafib illustrating the changes in peak intensities. (b) Chemical shift perturbations (CSPs) observed for PTP1B^1-301^ (5 molar excess of Ertiprotafib, *top panel*) and PTP1B^1-393^ (ten molar excess of Ertiprotafib, *bottom panel*), respectively.
